# High-metastatic cancer cells derived exosomal miR92a-3p promotes epithelial-mesenchymal transition and metastasis of low-metastatic cancer cells by regulating PTEN/Akt pathway in hepatocellular carcinoma

**DOI:** 10.1038/s41388-020-01450-5

**Published:** 2020-09-11

**Authors:** Beng Yang, Xiaode Feng, Hua Liu, Rongliang Tong, Jingbang Wu, Changbiao Li, Hanxi Yu, Yunhao Chen, Qiyang Cheng, Junru Chen, Xianlei Cai, Wenxuan Wu, Yuejie Lu, Jiating Hu, Kejiong Liang, Zhen Lv, Jian Wu, Shusen Zheng

**Affiliations:** 1 Division of Hepatobiliary and Pancreatic Surgery, Department of Surgery, The First Affiliated Hospital, Zhejiang University School of Medicine, Hangzhou, Zhejiang China; 2 NHC Key Laboratory of Combined Multi-organ Transplantation, Hangzhou, Zhejiang China; 3 Key Laboratory of the diagnosis and treatment of organ Transplantation, CAMS, Hangzhou, Zhejiang China; 4 Key Laboratory of Organ Transplantation, Hangzhou, Zhejiang China; 5 Department of gynecology and obstetrics, The First Affiliated Hospital, Zhejiang University School of Medicine, Hangzhou, Zhejiang China; 6 Department of orthopedics, The First Affiliated Hospital, Zhejiang University School of Medicine, Hangzhou, Zhejiang China

**Keywords:** Metastasis, Oncogenes

## Abstract

Exosomes play an important role in intercellular communication and metastatic progression of hepatocellular carcinoma (HCC). However, cellular communication between heterogeneous HCC cells with different metastatic potentials and the resultant cancer progression are not fully understood in HCC. Here, HCC cells with high-metastatic capacity (97hm and Huhm) were constructed by continually exerting selective pressure on primary HCC cells (MHCC-97H and Huh7). Through performing exosomal miRNA sequencing in HCC cells with different metastatic potentials (MHCC-97H and 97hm), many significantly different miRNA candidates were found. Among these miRNAs, miR-92a-3p was the most abundant miRNA in the exosomes of highly metastatic HCC cells. Exosomal miR92a-3p was also found enriched in the plasma of HCC patient-derived xenograft mice (PDX) model with high-metastatic potential. Exosomal miR-92a-3p promotes epithelial-mesenchymal transition (EMT) in recipient cancer cells via targeting PTEN and regulating its downstream Akt/Snail signaling. Furthermore, through mRNA sequencing in HCC cells with different metastatic potentials and predicting potential transcription factors of miR92a-3p, upregulated transcript factors E2F1 and c-Myc were found in high-metastatic HCC cells promote the expression of cellular and exosomal miR-92a-3p in HCC by directly binding the promoter of its host gene, *miR17HG*. Clinical data showed that a high plasma exosomal miR92a-3p level was correlated with shortened overall survival and disease-free survival, indicating poor prognosis in HCC patients. In conclusion, hepatoma-derived exosomal miR92a-3p plays a critical role in the EMT progression and promoting metastasis by inhibiting PTEN and activating Akt/Snail signaling. Exosomal miR92a-3p is a potential predictive biomarker for HCC metastasis, and this may provoke the development of novel therapeutic and preventing strategies against metastasis of HCC.

## Introduction

Hepatocellular carcinoma (HCC) is a deadly form of cancer and is the most common liver cancer worldwide [1]. Although some progress have recently been made in clinical diagnosis and treatment, late diagnosis and metastasis of HCC remain the main causes of high mortality, extremely threatening the long-term prognosis of HCC patients [2, 3]. Factors that promote malignancy and subsequent metastasis in HCC have not been fully elucidated. A comprehensive understanding of HCC metastasis and its underlying molecular mechanism can promote the development of potential therapeutic interventions against metastatic HCC.

Cancer is regarded as a disease resulting from clonal evolution in the body [4, 5]. From an evolutionary perspective, only after a series of genetic and epigenetic alterations can primary tumor cells form a tumor [6]. Cancer cells in a tumor can be viewed as a heterogeneous population of individual cells, including cancer-initiating cells and highly or less differentiated cancer cells with morphological and functional differences [7, 8]. Intratumor heterogeneity can arise from the somatic accumulation of mutations and cellular differentiation. Many studies have also revealed that intratumor heterogeneity could be induced by exerting selective pressures in the tumor. Intercommunication within cells or other factors in the microenvironment can foster the evolution of cancer cells.

Exosomes are a class of extracellular vehicles (30–150 nm diameter) that play a crucial role in cellular communication [9]. Most cells can secrete exosomes, which can be taken up by recipient cells to redirect their functions [10]. Intercellular communication is an important characteristic of tumor progression. Mounting evidence has pointed out that cancer exosomes or extracellular vesicles (EVs) participate in the regulation of epithelial to mesenchymal transition (EMT), which is a crucial step for cancer metastasis [11, 12]. And miRNAs are the most predominant RNA species in exosomes. In pancreatic cancer. Cancer-derived exosomal-miR-301 promotes EMT of pancreatic cells in hypoxic conditions [13]. Also, exosomal miRNAs result in PTEN loss and promote the metastasis of breast cancer in the brain [14]. Cancer exosomal miRNAs are also emerging as crucial messengers in cancer evolution and metastasis [15]. However, more investigations are needed to elucidate how cancer-derived EVs or exosomes regulate cell polarity and promote EMT in HCC [16].

## Results

### HCC cells with high-metastatic ability transmit invasion potential to low-metastatic HCC cells through exosomes both in vivo and in vitro

**Fig. 1:**
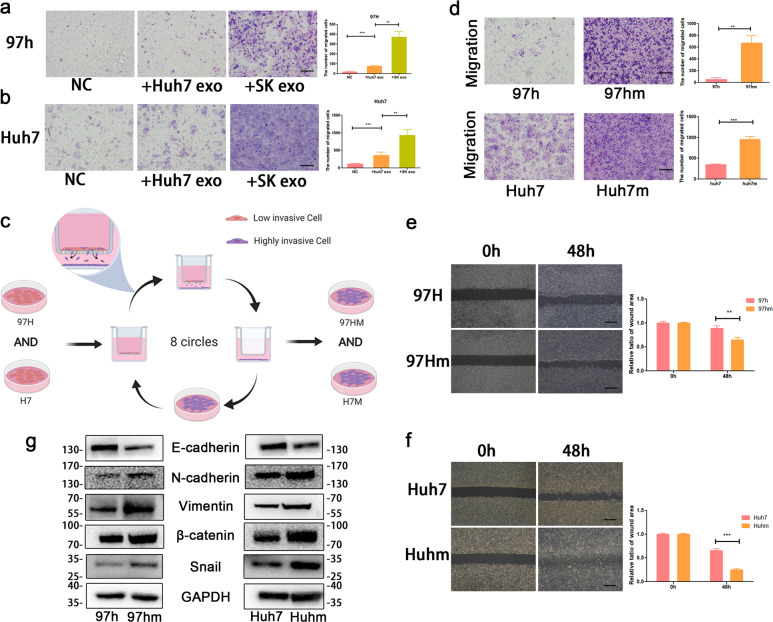
Construction of HCC cell lines with high-metastatic capacity (97 hm and Huh7). The effect of exosomes derived from Huh7 and SK cells on the migration ability of recipient cells (97 h and huh7 cells) (**a**, **b**). Schematic diagram of the method for obtaining 97hm and Huhm from MHCC-97h and Huh7 (**c**). Compared to 97h and Huh7, 97hm and Huhm exhibited more powerful motility after selection, measured by migration assay (**d**) and wound healing assay (**e**, **f**). Representative images are shown. Quantification is displayed via ImageJ software, and results are in triplicates. EMT associated proteins (including E-cadherin, N-cadherin, Vimentin, Snail, and beta-catenin) levels were detected by western blot (**g**). *P < 0.05, **P < 0.01, ***P < 0.001. Bars: (**a**) 100 μm; (**d**) 100 μm; (**e**–**f**) 200 μm.

Heterogeneity is pervasive in many aspects of cancer, including proliferation, metastasis, and drug resistance. First, to determine the metastatic heterogeneity of liver cancer cells, the migrating abilities of five liver cancer cells were examined by migration assays. Huh7 and SK-Hep-1 have more powerful migration abilities than HCCLM3, Hep-3B, and MHCC97H in vitro (Fig. S1a). The bioactive substance in the supernatant from cancer cells can regulate the phenotype and function of recipient cells. To verify our hypothesis that migration ability might be transferred among cancer cells through the bioactive substance in cell supernatant, conditioned media (CM) from Huh7, SK-Hep-1, and Hep-3B were utilized to study their effect on Huh7 cells. Huh7 cells, cultured with CM from relatively high-metastatic cells (Huh7 and SK-Hep-1 cells), yielded stronger migration ability than Huh7 cells cultured with CM from Hep-3B (Fig. S1b). The conditioned medium contained diffusible factors, including EVs, cytokines, and proteins [17–19]. To determine whether EVs alone can transmit metastatic merit to cancer cells, exosomes derived from Huh7 and SK cells were isolated and used to treat 97h and Huh7 cells, respectively. Cells treated with exosomes, especially from high-metastatic cancer cells, migrated more aggressive than the negative control cells (Fig. 1a, b).

These results revealed that different liver cancer cell lines could use exosomes to transmit migration ability to other liver cancer cells. Metastatic cancer cells derived from primary tumors are genetically homogeneous. Heritable variations in the mechanism of cellular communication during tumor evolution exist in different types, even subtypes of cancers [6, 20]. Therefore, malignant cellular communication among HCC cells are derived from homogenetic cells; this is representative of the reality of cancer in vivo. By continuously putting selective evolution pressure on 97 h and Huh7 cells, high-metastatic HCC cells (97 hm and Huhm) were obtained from primary cells (Fig. 1c). 97hm and Huhm showed a significant increase in migration ability, compared to the parental cells (Fig. 1d–f). And from the results of western blot, high-metastatic HCC cells (97hm and Huhm) have higher expression of mesenchymal associated proteins (N-cadherin, Vimentin, beta-catenin, and Snail) than their parental low-metastatic HCC cells (97h and Huh7) (Fig. 1g).

**Fig. 2:**
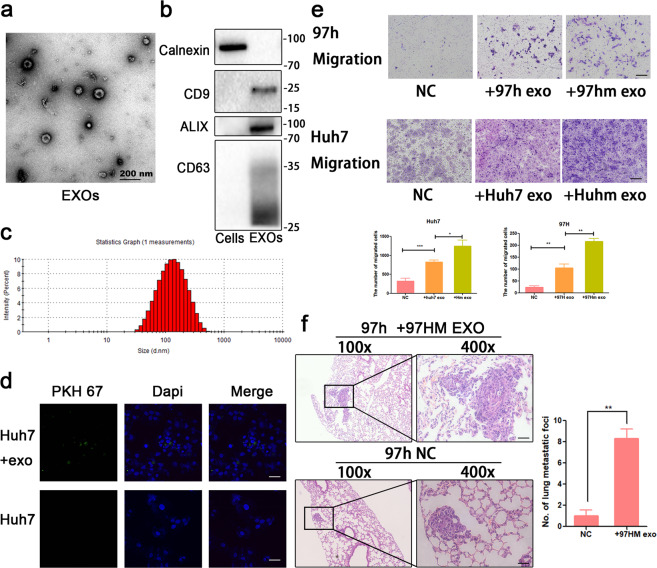
Exosomes derived from 97hm and Huhm promote metastasis of HCC both in vitro and in vivo. In the characterization of exosomes, they were firstly measured by transmission electron micrographic imaging (**a**), Western blot of Alix, CD9, CDD63 (typical exosomal markers), and Calnexin (negative control) (**b**) and Nanoparticle tracking assay (**c**). PKH67-labeled exosomes were added to be incubated with HCC cells and examined by confocal microscopy. Representative immunofluorescent images are shown with a magnification of ×400, demonstrating the successful uptake of exosomes by HCC cells (Green: exosomes, Blue: DAPI) (**d**). HCC exosomes, especially 97hm and Huhm exosomes, promote the migration ability of 97h and Huh7 HCC cell (**e**). Treatment with 97hm exosomes resulted in more metastasis in the HCC lung metastasis model compared to the negative control group. Representative HE images of lung metastasis are shown, and the number of lung metastasis per mouse was calculated (**f**). *P < 0.05, **P < 0.01, ***P < 0.001. Bars: (**a**) 200 nm; (**d**) 25 μm; (**e**) 100 μm; (**d**) left: 200 μm, right: 50 μm.

Next, exosomes from 97hm and Huhm were isolated via differential centrifugation and ultracentrifugation. The characteristic sizes and shapes of EVs were determined by transmission electron microscope (TEM) and nanosight tracking analysis (NTA), and western blot was used to characterize these exosomes and found the stable expression of typical exosomal marker proteins (Fig. 2a–c). Furthermore, PKH-67 labeled exosomes were treated with Huh7 cells, and green signals were observed in recipient cells after incubation for 24 h, indicating that high-metastatic HCC cell-derived exosomes can be absorbed successfully by the recipient HCC cells (Fig. 2d). Not only can 97hm- and Huhm-derived exosomes increase the migration ability of HCC cells in vitro (Fig. 2e), they can also facilitate HCC metastasis in vivo (Fig. 2f). These data demonstrate that high-metastatic HCC cells can endow “normal” HCC cells with more powerful metastatic abilities through secreted exosomes.

### High levels of miR-92a-3p are detected in exosomes of high-metastatic HCC

**Fig. 3:**
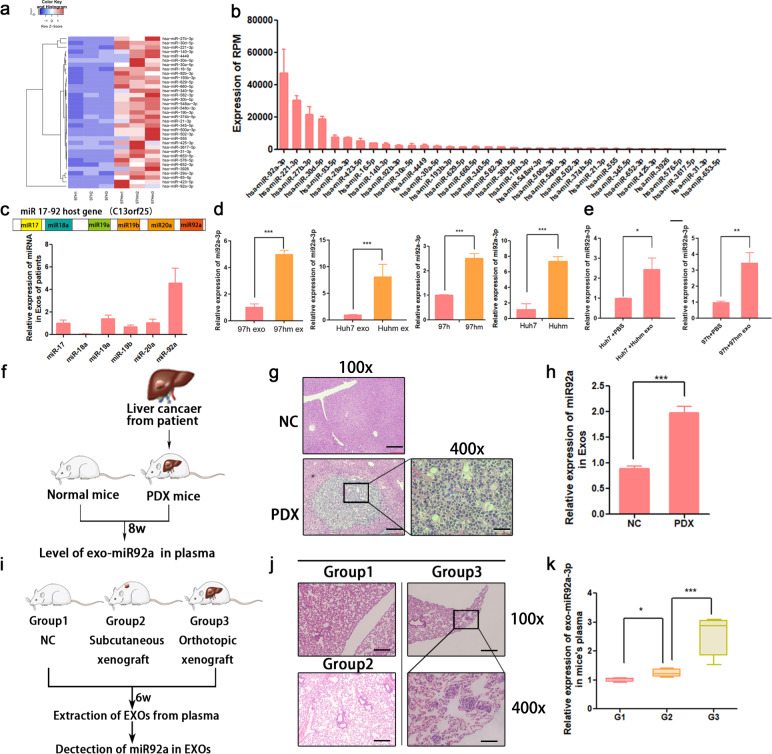
Cellular and exosomal miR92a-3p were upregulated in HCC cells with high-metastatic potential. Heatmap of differential exosomal miRs between 97h and 97hm were shown after miRs sequencing (**a**). Differential exosomal miRs were ranked by the level of miR’s expression, with the miR92a-3p being the most abundant differential miRNA (**b**). The expression level of miR17-92 family in plasma of HCC patients (**c**). Real-time PCR results show a significant overexpression of exosomal miR92a-3p and cellular miR92a-3p in 97hm and Huhm, compared with that in 97h and Huh7 (**d**). The cellular miR92a-3p levels of 97h and Huh7 were increased after incubation with exosomes from 97hm and Huhm (**e**). Tumor from HCC patient was implanted to construct a PDX mouse model (**f**). After 8 weeks, the PDX tumor was observed after HE staining (**g**) and exosomal miR92a-3p in plasma was measured by real-time PCR (**h**). Levels of exosomal miR-92a-3p were approximately twofolds higher in the plasma of the PDX group than that of the negative control group. Schematic diagram of HCC mice model of subcutaneous xenograft and orthotopic xenograft. After 6 weeks, tumors, lungs, and plasma were collected, *n* = 4 mice per group (**i**). Representative H&E staining images of lung section from each group (**j**). And the number of metastatic nodules were shown (**k**). *p < 0.05; ***p < 0.001. Bars: (**g**) left: 200 μm, right: 50 μm; (**j**) lower right: 50 μm, others: 200 μm.

Tumorigenic microRNAs encapsulated by exosomes play a pivotal role in cellular communication and tumor progression [21, 22]. To determine which RNAs were loaded in exosomes and served as main malignant messages, miRNA sequencing was performed among exosomal RNA samples derived from 97 h cells and its evolved 97 hm cells. The results show that 627 exo-miRNAs were upregulated in 97hm exosomes compared to 97h exosomes (Fig. s2). A heatmap of the most differentially expressed miRNA (*p* < 0.01) is depicted in Fig. 3a. Then differential miRNAs were ranked according to the level of expression, and miR92a-3p was the most abundant miRNA among the differentials (Fig. 3b). Furthermore, by using real-time q-PCR, we found that the levels of cellular and exosomal miR92a-3p in two high-metastatic HCC cell lines (97hm and Huhm) were higher than those in two “normal” HCC cells lines (97h and Huh7) (Fig. 3d). After incubating with exosomes from 97hm or Huhm, the cellular miR92a-3p expression in 97h and Huh7 cells increased significantly compared to the negative control group (Fig. 3e).

MiR-92a-3p is a member of the miR17-92 cluster family, correlating with poor prognosis in cancer patients, and other members of this oncogenic cluster are also found in 97hm exosomes [23]. To determine the potential role of these miRNAs as prognostic markers, the expression of these miRNAs was evaluated in plasma exosomes of HCC patients. According to the result of miRNA sequencing, miR92a-3p was the most abundant miRNA of this cluster in exosomes of high-metastatic HCC cells (Fig. 3c). Furthermore, plasma exosomal miR92a-3p increased significantly in HCC patient-derived xenograft (PDX) mice model than in normal mice (Fig. 3f–h). Next, to elucidate the potential role of miR92a-3p in metastasis, three HCC mice models were established: group 1; negative control, group 2; subcutaneous HCC xenograft (without lung metastasis), and group 3; orthotopic HCC xenograft (with lung metastases) (Fig. 3i, j). Exosomal miR92a-3p in plasma increased slightly in mice bearing subcutaneous HCC cancer (group 2), and increased obviously in the mice with metastases (group 3) compared to that in mice in group 1 (Fig. 3k). All these results indicate that the expression of exosomal miR92-3p increases with the development of HCC both in vitro and in vivo, thus, miR92a-3p may be an efficient marker for HCC progression.

### MiR92a-3p promotes invasiveness and malignancy of HCC

**Fig. 4:**
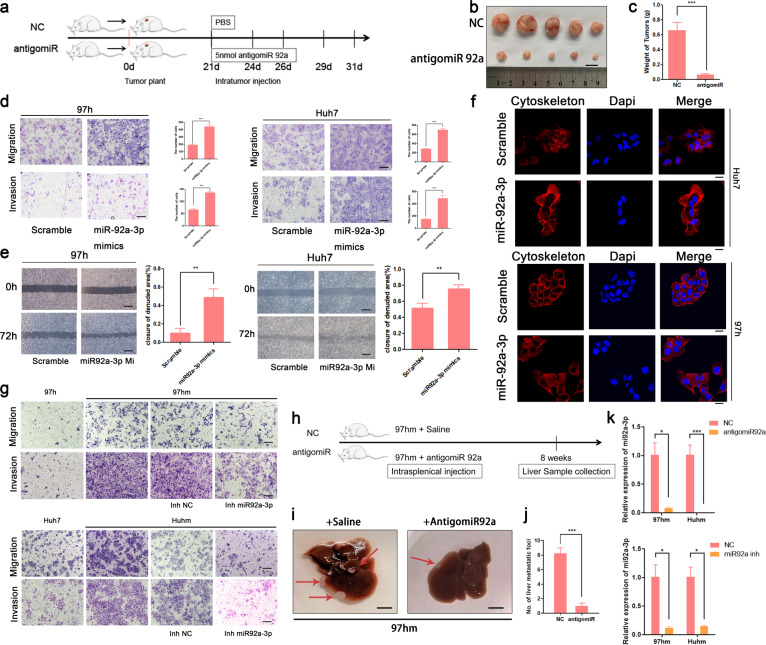
miR92a-3p promotes HCC metastasis both in vitro and in vivo. Schematic diagram of the mice model of HCC subcutaneous xenograft with the treatment of antigomiR92a-3p or PBS. 5nmol antigomiR92a3-3p or isopyknic PBS were injected into tumors every 2 or 3 days (**a**). Mice were sacrificed and tumors were harvested after 44 days. Optical images of tumors were photographed (**b**), and tumor volumes were recorded at indicated point times. Tumor volume growth curve(E) and tumor weight (**c**) of mice were shown, *n* = 5 mice per group, ***p < 0.001. The results of transwell assay showing the effect of overexpressed miR-92a-3p by miR-92a-3p mimics on migration and invasion ability of 97 h and Huh7 (**d**), ***P < 0.001. Wound healing assay also demonstrates a higher migration ability in miR-92a-3p overexpression group VS negative control group in 97h and Huh7 (**e**), **P < 0.01. Representative immunofluorescent images of cytoskeleton stained by phalloidin in Huh7 and 97h after transfection with miR92a-3p mimics or scramble mimics (**f**) (Images were magnified ×400). 97hm and Huhm obtained more powerful migration and invasion ability after the evolution based on 97h and Huh7. The inhibition of miR92a-3p decreases the enhanced metastatic abilities in these cells (**g**). 97hm was injected intravenously to construct a lung metastasis model, and antigomiR-92a-3p was used to decrease the level of miR-92a-3p. Schematic diagram of liver metastasis model of HCC with the treatment of antigomiR92a-3p was shown (**h**), Optical images of the lung with metastasis are shown; red arrows indicate tumor nodules (**i**). The numbers of metastases per lung were calculated (**j**). The expression of miR92a-3p of HCC cells after transfection with antigiomiR and inhibitor (**k**). *P < 0.05, **P < 0.01, ***P < 0.001 Bars: (**b**) and (**i**) 1 cm; (**d**) and (**g**) 100 μm; (**e**) 200 μm; (**f**) 10 μm.

The results above indicate that miR92a-3p was overexpressed in exosomes of HCC cell lines with a high-metastatic ability (97hm and Huhm), thus, promoting HCC metastasis both in vitro and in vivo. However, the exact role of miR92a-3p in HCC was not clear. MiR92a-3p was overexpressed exogenously via the transfection of miR92a-3p mimics in 97h and Huh7 cells (Fig. S3a). Experiments in vitro showed that miR92a-3p overexpression in HCC cells facilitated cell proliferation (Fig. S3c) and colony formation (Fig. S3b). Notably, when miR92a-3p was downregulated by antagomiR-92a-3p (Fig. 4k), tumor growth and progression of existing HCC tumors were suppressed significantly in vivo (Fig. 4a–c). Regarding cancer migration ability, upregulated miR92a-3p promoted migration and invasion ability both in 97h and Huh7 cells (Fig. 4d, e). Increasing cell mobility is a fundamental step for tumor invasion, which is accompanied by the regulation of the cell cytoskeleton [24, 25]. To determine the potential effect of upregulated miR-92a-3p on the cell cytoskeleton in HCC, phalloidin staining was performed. More microtubules and microfilaments were observed in the miR92a-3p overexpressed group, compared to that in the scramble group in HCC cells (Fig. 4f). High-metastatic HCC cells, 97hm and Huhm, with a higher expression of miR-92a-3p, were derived from 97h and Huh7 cells, respectively. Then, to identify the role of miR-92a-3p in HCC evolution, the expression of miR-92a-3p was decreased by a transfecting miR-92a-3p inhibitor (Fig. 4k). As Fig. 4g shows, 97hm and Huhm had a higher migration and invasion ability than did 97h and Huh7 cells, and the enhanced metastatic abilities were reduced by the downregulation of miR-92a-3p in 97hm and Huhm cells (Fig. 4g). Next, we wanted to explore whether miR92a-3p influences HCC metastasis in vivo. To achieve this, high-metastatic HCC cell lines (97hm) were used to construct liver metastasis models. Meanwhile, antigomiR92a-3p against miR92a-3p was injected via the tail vein. Our results show that the downregulation of miR92a-3p inhibits HCC metastases (Fig. 4h–j).

### MiR-92a-3p fosters HCC metastasis by promoting PTEN/Akt-mediated EMT

**Fig. 5:**
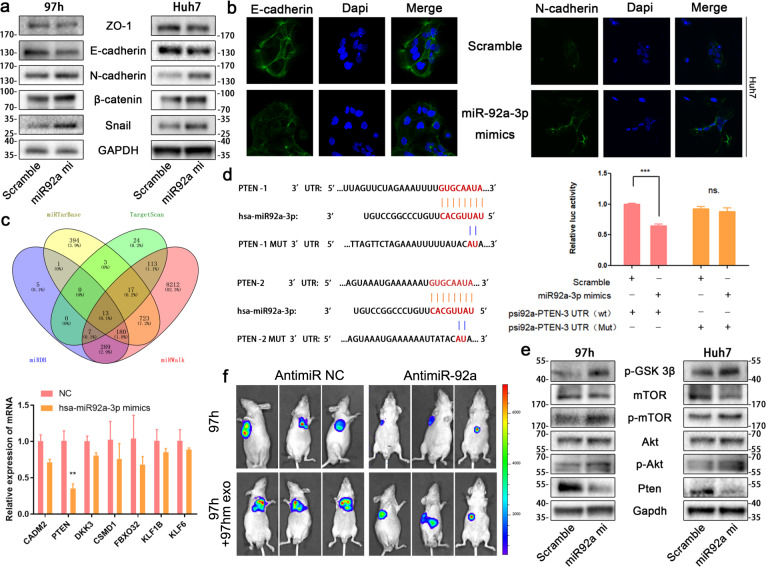
miR92a-3p promotes EMT by regulating Akt/Snail pathway via targeting PTEN. Western blot analysis showing the effect of upregulated miR-92a-3p on the protein level of ZO-1, E-cadherin, N-cadherin, β-catenin, and Snail in 97h and Huh7 (**a**). Representative immunofluorescent images of E-cadherin and N-cadherin in Huh7 was shown with a magnification of ×600 (Blue: DAPI, Green: E-cadherin or N-cadherin) (**b**). The downstream mRNA was predicted by four miRNA databases, and real-time PCR results showing a significant change of mRNA level (with PTEN being the most significant) in 97h cells after being transfected with miR92a-3p mimics or negative control mimics (**c**). Sequence alignment between miR-92a-3p and PTEN mRNA shows two mRNA segments of PTEN match with the seed sites of miR92a-3p, and nucleotide substitution mutation of the seed sites of PTEN constructed accordingly, and Luciferase reporter gene assay was performed in triplicate in 97H cells. Inhibitory luciferase activities were observed after transfection of miR92a-3p mimics, representative data were shown in the bar graphs, ***P < 0.001 (**d**). Western blot results show the changing protein level of p-GSK 3β, mTOR, Akt, PTEN, phosphorylated mTOR, and phosphorylated Akt in HCC after transfected with miR mimics (**e**). 97h-luciferase cells were used to construct lung metastasis models with different treatments. Representative bioluminescent imaging of mice was performed after 8 weeks, and luciferin was intraperitoneally injected via the IVIS spectrum. The image of the metastatic condition of mice with different treatments was shown, *n* = 4 mice per group (**f**). Bars: (**b**) 10 μm.

The initiation of cancer metastasis is greatly attributed to EMT in cancer cells. We wanted to explore whether miR92a-3p is involved in the regulation of EMT in HCC. In this case, Western blots and immunofluorescence were performed to detect the expression of EMT biomarkers. As expected, overexpression of miR92a-3p increased the expression of mesenchymal biomarkers (N-cadherin, β-catenin, Snail), and decreased the protein level of E-cadherin in the meantime (Fig. 5a, b, Fig. S4). To identify the target of miR92a-3p, four databases (miRDB, miRWalk, TargetScan, and miRTarBase) were utilized to predict the potential targets of miR92a-3p, and real-time q-PCR was used to verify the mRNA expression of 13 selected genes after miR92a-3p overexpression; only 7 genes were downregulated significantly with PTEN decreasing most obviously (Fig. 5c). Actually, western blot was performed to detect the protein expression of CADM2, PTEN, and DKK3, and only the expression of PTEN protein reduced significantly after overexpression of miR92a-3p (data not shown). Hence, it can be deduced that miR-92-3p downregulated PTEN in HCC. Then, a Dual-luciferase reporter plasmid system, which contained 3′-UTR of PTEN mRNA, was constructed (Fig. 5d). Luciferase reporter plasmids and miR92a-3p mimics or scramble mimics were co-transfected into 97h cells, and luciferase activities were detected. Results show that miR92a-3p interacted with PTEN 3′ UTR directly and decreased the expression of PTEN (Fig. 5d). PTEN was regarded as a crucial regulator of Akt [26], which is of great importance for EMT and cancer metastasis. Through Western blot, miR92a-3p was found inhibited the expression of PTEN, followed by the upregulation of Phosphorylated Akt, Phosphorylated mTOR, and Phosphorylated-GSK-3β (Fig. 5e). Next, to determine the effect of exosomal miR-92a-3p derived from 97hm in promoting metastasis of HCC in vivo, a liver metastasis model was constructed by injecting 97h-luciferase cells via the spleen vein. Exosomes derived from 97hm were injected via the tail vein to promote lung metastasis, and antigomiR-92a-3p was used to reduce the level of miR-92a-3p in HCC. AntigomiR-92a-3p reduced the HCC metastasis caused by exosomes (Fig. 5f).

**Fig. 6:**
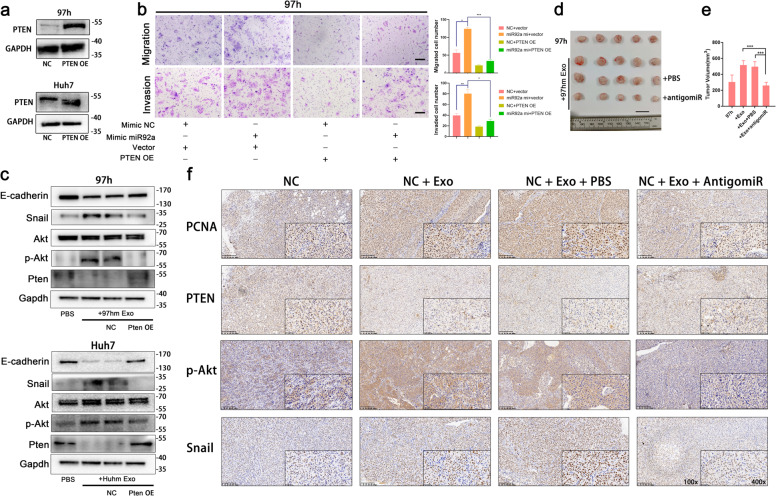
High-metastatic HCC-derived exosomes mediated EMT in HCC were neutralized by miR92a-3p inhibition and PTEN overexpression. PTEN was overexpressed in 97h and Huh7 cells by by PTEN-OE plasmids (**a**). The results of migration and invasion assay of 97h were shown after transfected with miR92a-3p or NC mimics and PTEN overexpression or vector plasmids. The incubation time of migration assay and invasion assay are 72h, 92h (**b**). After co-incubation with 97hm or Huhm exosomes or not, western bolt results show the changing protein level of E-cadherin, Snail, Akt, phosphorylated Akt and PTEN in 97h and Huh7 cells with the transfection of NC and PTEN-OE plasmids (**c**). Subcutaneous HCC xenograft model with the treatment of 97hm exosomes and antigomiR92a-3p was used. Mice were sacrificed and tumors were harvested after 35 days. Optical images of tumors were photographed, *n* = 5 mice per group (**d**). Tumor volumes were recorded and calculated (**e**). The IHC staining of PCNA, PTEN, p-Akt, and Snail in xenograft tumor of mice with different treatments were showed (**f**). *p < 0.05; **p < 0.01, ***p < 0.001. Bars: (**b**) 100 μm (**d**) 1 cm (**f**) Left: 200 μm; Right: 50 μm.

Notably, once PTEN was overexpressed by plasmid (Fig. 6a), enhanced migration and invasion abilities in HCC induced by miR92a-3p overexpression were reverted to the normal levels (Fig. 6b and Fig. S5). And the result of western blot showed the downregulation of PTEN and E-cadherin and upregulation of phosphorylated Akt and Snail caused by exosomes treatment was restored to basal level after PTEN overexpression plasmid were treated in HCC cells in vitro (Fig. 6c). Next, to elucidate whether miR-92a-3p influence PTEN/Akt pathway in vivo, a subcutaneous tumor xenograft model was used. 97hm exosomes treatment promoted tumor growth, and this promotion was neutralized by the treatment of antigomiR92a-3p (Fig. 6d, e). Tumors were collected for immunohistochemistry (IHC). From the result of IHC, the expression of PTEN was reduced and phosphorylated Akt and Snail protein levels were upregulated after treatment of high-metastatic HCC-derived exosomes. Once miR92a-3p in tumor xenograft was neutralized by antigomiR92a-3p, PTEN, p-Akt, and Snail level were restored to basal level in vivo as well (Fig. 6f). These data indicate that miR92a-3p promotes EMT via regulating PTEN/Akt pathway and play a crucial role in metastasis of HCC.

### Transcription factors c-Myc and E2F1 promote cellular and exosomal miR-92a-3p in HCC

**Fig. 7:**
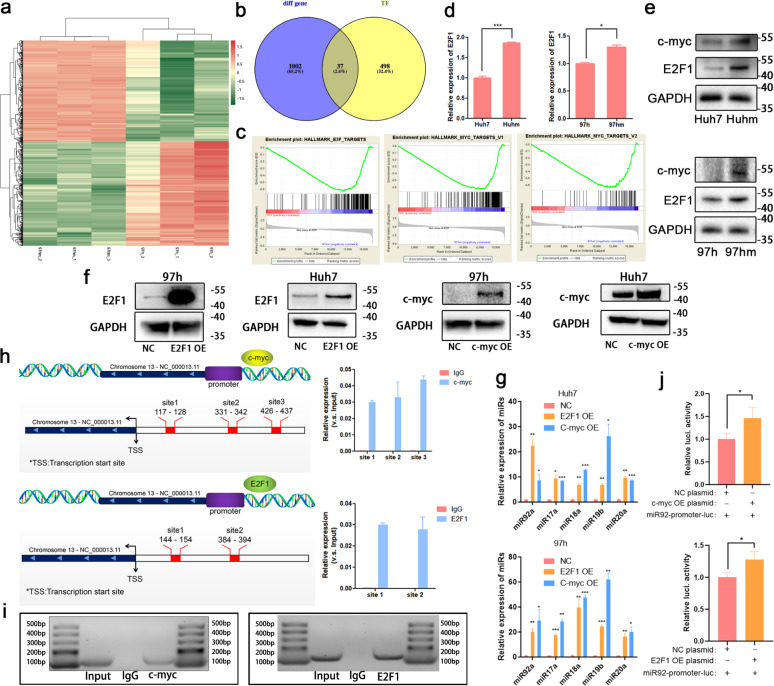
Transcription factors E2F1 and c-Myc promote the expression of miR-92a-3p. Heatmap of differential mRNAs between 97h and 97hm after mRNA sequencing (**a**). Overlapping results among the differential mRNA from mRNA sequencing and potential directly transcription factors of miR92a-3p predicted by JASPAR database (**b**). GO (Gene Ontology) enrichment analysis of E2F1 and c-Myc related signaling in 97h and 97hm (**c**). E2F1 and c-Myc were found to be overexpressed in 97hm and Huhm compared to 97h and Huh7. The results of PCR were normalized with house-keeping gene GAPDH, *P < 0.05 ***P < 0.001. Analysis of the mRNA level of the indicated gene was determined by real-time PCR (**d**), and the protein level of E2F1 and c-Myc were determined by Western blot (**e**). E2F1 and c-Myc overexpression plasmids were transfected into HCC cells (**f**). The level of miR17-92 cluster members in TFs OE group and negative control group were determined by Real-time PCR (**g**). E2F1 and c-Myc induced the expression of miR92a-3p. The diagram showing the binding sites of E2F1 and c-Myc in the specific sequence of the host gene of miR92a-3p (miR17HG) and the relative expression of segments containing E2F1 or c-Myc binding sites was detected by real-time PCR after ChIP assay (**h**). Northern blot of indicated segments after ChIP assay with antibodies anti-c-Myc and anti-E2F1, respectively (**i**) Relative luciferase activity of miR17HG (including miR92a-3p promoter) in 97h after the co-transfection of TFs overexpression or negative control plasmids and miR92a-promoter-luciferase plasmids (**j**).

An increase in cellular and exosomal miR92a-3p levels accompany the enhancement of metastatic capacity in HCC cells. Mounting evidence indicates that transcription factors (TFs) can trigger and promote metastasis [27, 28], and regulate the expression of multi miRNAs or protein in cancers as well. To ascertain the potential TFs that are responsible for the upregulation of miR92a-3p, mRNA sequencing between 97h cells and 97hm cells was performed (Fig. 7a). Moreover, intersection analysis of the above mRNA sequencing results and potential transcription factors predicted by the JASPAR database (http://jaspar.genereg.net/) showed that 37 candidates might directly promote the expression of miR92a-3p (Fig. 7b). After GSEA analysis among these candidates, E2F1 and c-Myc related pathways are found greatly enriched in 97hm cells compared to that in 97h cells (Fig. 7c). It is a consensus that the carcinogenic effects of E2F1 and c-Myc, and E2F1 is expressed in a variety of cancers (Fig. S6a). In particular, through the GEPIA database (http://gepia.cancer-pku.cn/), a high expression level of E2F1 was found associated with high TNM stage of HCC, indicating a poor prognosis of HCC patients (Fig. S6b, c). What’s more, the mRNA and protein expression of these two TFs were also found increased in 97hm and Huhm, compared to those in 97H and Huh7 (Fig. 7d, e). Moreover, Overexpression of E2F1 and c-Myc promoted the expression of miR92a-3p and other members in miR17-92 cluster in HCC cells (Fig. 7f, g). To verify the putative c-Myc binding sites (CBSs) and E2F1 binding sites (EBSs) in the promoter of *miR17HG*, the host gene of miR-92a-3p, chromatin immunoprecipitation (ChIP) assays were performed. As Fig. 7h shows, there were three CBSs and two EBSs; CBS1 and EBS1 were further confirmed by Southern blot assay (Fig. 7i). Then, *miR92*-promoter-luciferase reporter plasmid system was constructed, and it was co-transfected with E2F1 and c-Myc overexpression plasmids into 97h cells. Luciferase activities were detected after transfection. As Fig. 7j shows, E2F1 and c-Myc can both activate the expression of miR92a-3p. All these demonstrate that the E2F1 and c-Myc directly bind the specific DNA sequences of *miR17HG* and promote the expression of miR92a-3p in HCC cells.

### Exosomal miR-92a-3p in plasma of HCC patients correlates with cancer metastasis

**Fig. 8:**
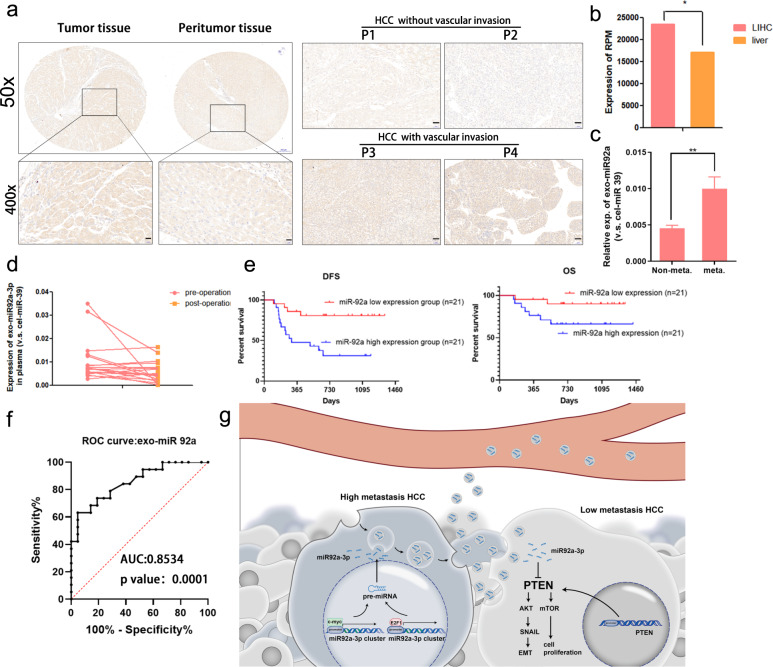
MiR-92a-3p level is associated with lung metastasis and cancer progression in HCC. In situ hybridization of miR-92a-3p in the section of tumor tissue and its corresponding peritumor tissue from HCC patient, and the resulting in situ hybridization of miR-92a-3p revealed a higher level of miR92a-3p in the tumor of HCC patient with vascular invasion than those without vascular invasion. The miR-92a-3p expression level of HCC tumor and normal liver tissues from GEPIA database, *P < 0.05 (**b**). Exosomal miR92a-3p expression level in plasma from 42 HCC patients with or without metastasis. Non-meta., HCC patients without metastasis; Meta., HCC patients with metastasis, **P < 0.01 (**c**). The exosomal miR-92a-3p in plasma of HCC patients at different timepoints were measured (pre-operation, before liver operation; post-operation, 7 days after the liver operation). The consecutive change of miR92a-3p level was shown in the graph (**d**). Correlation between the levels of exosomal miR92a-3p in plasma with the overall survival rate and diseases free survival rate of 42 HCC patients (**e**). Kaplan–Meier method and the log-rank test were used to analyze survival data. ROC curve for exosomal miR-92a-3p in plasma to discriminate primary HCC patients without from HCC patients with metastasis (**f**). **g** Schematic diagram of how E2F1 and c-Myc mediated exosomal miR92a-3p in high-metastatic HCC cells regulate PTEN expression to promote EMT and HCC metastasis. Bars: (**a**) 200 μm; (**j**) 50 μm.

To determine the role of miR92a-3p in HCC patients, in situ hybridization was used to detect the expression of miR92a-3p in HCC tissues and its corresponding peritumor tissues. As shown in Fig. 8a, elevated miR92a-3p was detected in HCC tissues, compared to that in peritumor tissues, which is consistent with the data from the GEPIA database (Fig. 8b). More importantly, higher-levels of miR-92a-3p were seen in HCC tissues with vascular invasion, compared to HCC without vascular invasion (Fig. 8a). Next, the expression of exosomal miR-92a-3p was investigated in plasma samples of 42 HCC patients (21 HCC without metastasis, 21 HCC with metastasis). The levels of exosomal miR-92a-3p in the plasma of HCC patients with metastases were significantly higher than in HCC patients without metastasis (Fig. 8c). In addition, the relationship between exosomal miR-92a-3p expression levels and the clinicopathologic features of 42 HCC patients was analyzed and listed in Table 1. Hepatectomy is a common and effective therapy for most cases of HCC. Thus, to further understand the correlation between miR92a-3p expression and disease progression, preoperative plasma samples and postoperative plasma samples (7 days after surgery) were collected from patients with primary HCC and used to detect exosomal miR92a-3p expression. And as Fig. 8d shows, exosomal miR92a-3p levels decreased after the tumor was removed via surgery. HCC patients were assigned into two groups based on the level of exosomal miR92a-3p in plasma (miR92a-3p high group and miR92a-3p low group), and high expression of exosomal miR92a-3p was correlated with low overall survival and poor disease-free survival, indicating poor prognosis in HCC patients. (Fig. 8e). Furthermore, The AUC-ROC for exosomal miR92a-3p in comparison between HCC patients without metastasis and HCC patients with metastasis was 0.8534 (*P* < 0.001, Fig. 8f).

**Table 1:** Clinical characteristics of 42 HCC patients depending on exosomal miR92a-3p levels in plasma.

Variable	Exosomal miR92a-3p level		*N*	*P*-value
	Low-miR92a	High-miR92a		
All cases	21	21	42	
Age (years)				
≦55	15	8	23	0.062
>55	6	13	19	
Gender				
Male	18	20	38	0.303
Female	3	1	4	
Tumor size (cm)				
≤5	15	14	29	0.5
>5	6	7	13	
Number of tumor nodules				
1	7	7	14	0.628
>1	14	14	28	
Tumor differentiation				
1–2	12	10	22	0.379
3–4	9	11	20	
AFP (ng/ml)				
≤400	15	13	28	0.372
>400	6	8	14	
Vascular or lymph node invasion				
Yes	6	13	19	0.031^*^
No	15	8	23	
Recurrence				
Yes	5	15	20	0.002^*^
No	16	6	22	

Tumor differentiation 1–4 in pathology diagnosis is equivalent to normal tissue, well differentiated, moderately differentiated, poorly differentiated or undifferentiated, respectively.

*AFP* alpha fetoprotein.

*Statistically significant.

Summarily, exosomal miR-92a-3p was overexpressed in HCC cells with high-metastatic capacity and HCC patients with metastases. HCC-derived exosomal miR-92a-3p converted “normal” HCC cells to more aggressive HCC cells via mediating the reduction of PTEN and activating the Akt/Snail signaling pathway to promote EMT and metastasis in HCC (Fig. 8g). Our results also indicated that HCC-derived exosomal miR-92a-3p involved in the progression of HCC, plays a pivotal role in intercellular communication and promoting HCC metastasis, and that exosomal miR-92a-3p could be a novel biomarker of bio-liquid biopsy in metastatic HCC.

## Discussion

Primary liver cancer is a lethal disease, for which the associated 5-year survival rate is <20%. Metastasis and recurrence are the major causes of cancer-related deaths [29]. Cellular communication in cancer cells plays an important role in cancer progression, and this promotes cancer evolution in many aspects, including drug resistance, metastasis, and recurrence. Cancer parenchymal cells make up the most part of the neoplasm and due to the existence of intratumor heterogeneity and the cell viability, metastatic and drug-resistant abilities of heterogeneous cancer cells vary from subgroup to subgroup. Therefore, it is necessary to understand the relationship between intercellular communication and cancer evolution, and identify the underlying mechanism and potential therapeutic targets of metastasis in HCC.

Cancer heterogeneity, including intertumor heterogeneity and intratumor heterogeneity, exist in each stage of tumorigenesis. Intratumor heterogeneity is ubiquitous in cancers, and it is at the base of clonal evolution and cancer evolution [30]. According to Alvin Makohon-Moore, within a pancreatic ductal adenocarcinoma, genotypic heterogeneity drives phenotypic heterogeneity, which can help cancer cells to survive [31]. In addition, the heterogeneity of non-parenchymal cells within tumors is also involved in the progression of cancer in HCC [32].

Cancer is a complexity of cell subpopulation with different phenotypes and functions, arising from some simple evolutionary processes, including mutation, genetic selection, and numerous interacting agents. It is the change caused by selective pressures over time that drives cancer promotion and leads to adaptation; that is, the selection is the key for cancer evolution [33]. So, to mimic the natural process in the evolution of tumor metastatic ability, high-metastatic HCC cells were constructed by exerting selective pressures on primary HCC cells. Next, the different miRNA profiles from the exosomes of high-metastatic HCC cell (97hm) and its primary HCC cell (MHCC-97h) were analyzed. MiR-92a-3p was found upregulated both in the cytoplasm and exosomes of HCC cells with the enhancement of metastatic ability. Then, we identified exosomal miR92a-3p that was transferred to recipient cancer cells to promote EMT, which facilitates the metastasis of HCC by suppressing PTEN to activate the Akt/Snail signaling pathway. To some extent, the crosstalk between heterogeneous cancer cells with different metastatic potential in HCC elucidates the molecular mechanism of cancer evolution and progression in metastasis.

According to the Neo-Darwinian synthesis of evolution biology, at the center of the evolution of an organism is the molecular evolution, especially the inherited change of DNA [34]. Thus, to find novel metastasis-associated biomarkers and potential therapeutic targets, the underlying mechanism of HCC metastasis needs to be explored. In this study, we demonstrated that transcription factors E2F1 and c-Myc upregulated the progression and metastatic ability of cancer. E2F1 and c-Myc drive the expression of miR92a-3p in HCC by directly binding to the promoter of *miR17HG*, the host gene of miR-92a-3p, thus promoting the progression of HCC. However, c-Myc can act as an oncogene or a promoter of apoptosis in various tumors, depending on its binding and regulating molecules. The exact role of E2F1 and c-Myc in the metastasis of HCC and their driving factors during evolution needs to be further explored.

As a member of miR-17-92 cluster, previous studies have shown that miR92a-3p is aberrantly expressed and exerts various effects in multiple cancers. For instance, miR92a-3p is secreted by tumor cells and stimulates tumor-associated macrophages to produce IL6, a pro-inflammatory cytokine, subsequently promoting the progression of liposarcoma [35]. MiR92a-3p also plays its oncogenic role in gastric cancer [36], colorectal cancer [37] and breast cancer [38]. However, miR92a-3p may have other roles in cancers. MiR-92a-3p was found to be overexpressed in human glioma samples and increased tumor malignancy and tumorigenesis, however, miR-92a-3p could also inhibit the stemness of glioma stem-like cells [39]. Our data demonstrate that HCC-derived exosomes transport miR-92-3p to recipient HCC cells, promote EMT, and convert low-metastatic HCC cells into high-metastatic HCC cells. In addition, high expression of exosomal miR-92a-3p in plasma is positively correlated with the metastasis of HCC, indicating a poor prognosis of HCC patients. Thus, treatments targeting miR-92a-3p may provide an efficient therapeutic option against HCC.

Recently, liquid biopsy has served as a powerful diagnostic method for many diseases, including cancers [40]. Through the collection of blood or other nonsolid body fluids from patients and analyzing its contained circulating cancer cells, cell-free DNA, and exosomes, liquid biopsy could accurately and dynamically diagnose cancers [41]. However, due to the low number and short survival time of circulating tumor cells (CTCs), it is hard to capture CTCs in the early stages of cancer [41]. However, due to the low number and short survival time of CTCs, it is hard to capture CTCs in the early stage of cancer [42, 43]. So, the clinical application of CTCs in the early detection of cancer progression is still challenging. However, exosomes are widespread in body fluids and can dynamically reflect the progression of tumors. For example, according to previous studies, serum exosomal miR-93, miR-21, and mi-9-3p can serve as effective indicators of HCC [44–46], and exosomal miR-103 and miR-1247-3p in the serum were related to a high risk of recurrence and metastasis in HCC patients [47, 48]. In our study, the level of exosomal miR92a-3p in plasma was detected and found to be decreased after tumor resection, depending on the HCC variant. In addition, high expression of exosomal miR92a-3p was positively correlated with the metastasis of HCC, suggesting that exosomal miR-92a-3p could be an effective and dynamic diagnostic biomarker for HCC.

In conclusion, high-metastatic HCC-derived exosomes transmitted metastatic potential to recipient cancer cells via transferring miR-92a-3p. MiR-92a-3p activated Akt/Snail pathway promotes the EMT and tumorigenesis of HCC via selectively suppressing tumor suppressor gene PTEN. In addition, E2F1 and c-Myc were upregulated, efficiently and directly driving the expression of miR92a-3p in HCC, thus leading to an increase in metastatic tendencies. More importantly, the expression of exosomal miR-92a-3p in plasma shows a positive correlation with metastasis in HCC patients. Our results are consistent with those of other studies, which indicated that miR-92a-3p is a promoter of cancer progression, and we are the first to systematically illustrate the relationship between exosomal miR-92a-3p and metastasis in HCC. This study elucidates the important role of intercellular communication among heterogeneous cancer parenchymal cells. It reveals a novel molecular mechanism involved in promoting metastasis in HCC, which can contribute to the generation of efficient early detection and new therapeutic strategies for metastatic HCC.

## Conclusion

Hepatoma-derived exosomal miR92a-3p plays a critical role in the progression of EMT and promotion of metastasis by inhibiting PTEN and activating Akt/Snail signaling pathway. Exosomal miR92a-3p is a potential predictive biomarker for HCC metastasis. This knowledge may spur the development of novel therapeutic and preventive strategies against metastasis of HCC.

## Materials and methods

### Human tissue and blood samples

With informed consent from all patients, all samples, including tissue and plasma specimen, were collected from the First Affiliated Hospital of Zhejiang University. Blood sample (plasma) from 21 primary HCC patients with metastasis and 21 primary HCC patients without metastasis were collected and stored at −80 °C. This study follows the ethical guidelines of the 1975 Declaration of Helsinki and protocols were approved by the Ethics Committee of the First Affiliated Hospital of Zhejiang University.

### Cell viability detection

Cell Counting Kit-8 (CCK-8) assay was performed as previously described to evaluate the cell viability [49].

### Western blotting

Cells or exosomes were collected and lysed in the RIPA Lysis buffer (Beyotime Biotechnology, China) containing Protease Inhibitor Cocktail (ThermoFisher, USA). Protein concentration was measured by Bradford assay (Bio-Rad Laboratories, Inc., Hercules, USA). Western blots were performed as previously described [50].

### Statistical analysis

All data were presented as the mean ± SD. The SPSS 22.0 software (IBM, NY, USA) was used for statistical analyses. Comparisons between two groups were assessed using a two tailed Student’s *t*-test. The Kaplan–Meier method was used to assess the overall survival rate of patients. *P* < 0.05 was considered as statistically significant difference.

All primers sequences are listed in Table 2. Other methods and materials are in the Supplementary information, which is available at oncogene’s website.

**Table 2:** The sequence of primers.

Name	Forward primer (5′--3′)	Reverse primer (5′--3′)
E2F1	ACGCTATGAGACCTCACTGAA	TCCTGGGTCAACCCCTCAAG
C-MYC	GGCTCCTGGCAAAAGGTCA	CTGCGTAGTTGTGCTGATGT
PTEN	TGGATTCGACTTAGACTTGACCT	GGTGGGTTATGGTCTTCAAAAGG
GAPDH	GGAGCGAGATCCCTCCAAAAT	GGCTGTTGTCATACTTCTCATGG
DKK3	AGGACACGCAGCACAAATTG	CCAGTCTGGTTGTTGGTTATCTT
FBXO32	GCCTTTGTGCCTACAACTGAA	CTGCCCTTTGTCTGACAGAAT
KLF1B	ATGTCGGGAGCCTCAGTGAA	GCATTTGGATTCCTTGCTGGT
CADM2	AAACTTCCAAGGCATATCTCACC	TGCGATTTGCATCCTCTTCTT
CSMD1	TGGAGGAGATTCCAGTCGCT	GCATAGTTCGGATACCCGTGA
KLF6	GGCAACAGACCTGCCTAGAG	CTCCCGAGCCAGAATGATTTT
Cel-miR-39	TCACCGGGTGTAAATCAGCTTG	
hsa-miR-92a-3p	TATTGCACTTGTCCCGGCCTGT	
hsa-miR-19b-3p	TGTGCAAATCCATGCAAAACTGA	
hsa-miR-17-5p	CAAAGTGCTTACAGTGCAGGTAG	
hsa-miR-19a	TGTGCAAATCTATGCAAAACTGA	
hsa-miR-18a	TAAGGTGCATCTAGTGCAGATAG	
hsa-miR-20a	TAAAGTGCTTATAGTGCAGGTAG	

## Supplementary information

supplement material
