# Supplementary material for: High-metastatic cancer cells derived exosomal miR92a-3p promotes epithelial-mesenchymal transition and metastasis of low-metastatic cancer cells by regulating PTEN/Akt pathway in hepatocellular carcinoma
Source: Oncogene. 2020 Sep 11;39(42):6529–43. doi: 10.1038/s41388-020-01450-5 (PMC7561497; doi:10.1038/s41388-020-01450-5)
Supplement: Supplementary file 1 — supplement material [file 41388_2020_1450_MOESM1_ESM.docx]

**Methods and Materials**

**Cell culture**

Human hepatocellular carcinoma cell line HCCLM3, Hep-3B, MHCC97H, Huh7, SK-Hep-1 were purchased from Cell Bank of the Chinese Academy of Sciences (Shanghai Institute of Cell Biology), all these cells as well as established high metastatic HCC cell lines (97hm and Huhm) were cultured in Dulbecco’s modified Eagle medium(DMEM) (Biological Industries, Israel) supplemented with 10% fetal bovine serum (FBS)(Moregate Biotech, Australia) at 37 ℃ in a humidified chamber with 5% CO2.

**Establishment of 2 HCC cells lines with high metastatic ability**

To select and induce generation of high metastatic HCC cells, MHCC97H and Huh7 cell line were digested and seeded into the upper chamber of Transwell (24-well plate, 8 μm pore size, Millipore, USA), cultured in 200μL DMEM without FBS. 800 μL DMEM with 10% FBS were used to fulfill lower chamber. After 48 hour’s culture, migrated cells were digested, collected and named as MHCC97H m1 and Huh7 m1, these cells were cultured for the next round operation. Repeat above operation 8 times, MHCC97H m8 and Huh7 m8 (High metastatic HCC derived from MHCC97H and Huh7) were obtained, and for convenience, they were called 97Hm and Huhm in the following research.

**Conditioned Media Preparation**

Hep-3B, Huh7, SK-Hep-1, MHCC-97h, 97hm and Huhm cells with a Confluence of 70%~80% were incubated with DMEM without FBS. After two days, the conditioned media was collected, filtered by a 0.22 μm size filter (Millipore, USA) for further use.

**Exosomes Preparation and Characterization**

Cell culture medium was collected and centrifuged at 300g for 5 min, followed by 2000g for 15min to remove cells and debris. The supernatant was centrifuged at 10000g for 45min to pellet cellular debris and large EVs. The supernatant was collected and filtered with a 0.22 μm size filter (Millipore, USA), followed by ultracentrifugation (Beckman, USA) at 140000g for 70 min at 4℃ to pellet exosomes. These exosome pellets were washed with 35ml PBS for one time and regained by ultracentrifugation at 140000g for 70 min at 4℃. The exosomes were resuspended in PBS and the protein concentration of exosomes was quantified by Bradford assay (Bio-Rad Laboratories, Inc., Hercules, USA). Exosome was utilized immediately or stored in −80 °C for future use. In all *in vitro* assays, 5µg/ml exosome was premixed with culture medium with FBS before use. As for all *in vivo* assays, the using dosage of exosomes was 20ug exosomes per mice. Transmission electron microscope (TEM) and Multi-parameter nanoparticle optical analysis (Nanosight) were used to determine the size, shape of exosomes. Antibodies anti-CD63 (ab59479, Abcam, MA, USA) and anti-CD9(ab92726, Abcam, MA, USA) were used to recognize EVs specific proteins by Western blot. And anti-Calnexin antibody (2679, CST, MA, USA) was utilized to perform western blot as a negative control.

**Detection of exosome transfer**

Isolated Exosomes were labeled with PKH67 by using PKH67 Fluorescent Cell Linker kits (SigmaAldrich, MO, USA) according to the instructions of manufacturer. PKH67-labeled exosomes were resuspended in DMEM medium with 10% FBS and co-culture with a subconfluent layer of HCC cells for 12 hours in a humidified incubator at 37℃. After incubation, cells were washed by PBS three times. Then, cells were fixed with 4% paraformaldehyde for 30 min. After washing with PBS three times, cells were further stained by DAPI (4, 6-diamino-2-phenylindole) for 10 min and observed under confocal laser scanning microscope (CLSM, IX82, Olympus, Japan).

**Exosomal miRNA sequencing and cellular mRNA sequencing**

The exosomal miRNA-Seq experiment was performed by RiboBio company (GuangZhou, China). Briefly, approximate 50 ng total exosomal RNA was used to construct a library using the NEBNext Multiplex Small RNA Library Prep Set for Illumina (Illumina, San Diego, CA) according to the manual of manufacturer. Libraries were then amplified and sequenced using HiSeq Rapid SBS Kit V2 (50 cycles) and HiSeq Rapid SR Cluster Kit V2 at the HiSeqTM 2500 system (Illumina).

As for mRNA sequencing, total RNA of 97h and 97hm were extracted by using TRIzol reagent following the instruction of manufacturer, mRNA was purified from total RNA using magnetic beads with Oligo (dT), and fragmented into ~200 bp short fragments, and then cDNA libraries were constructed. RNA-seq was performed on the Illumina HiSeq 2000 platform according to the manufacturer's instructions and reads were generated.

**miR Transfection and plasmids transfection**

The miR mimics and inhibitor used in this study were listed as follows: The micrONTM hsa-miR-92a-3p mimic (Ruibo biotechnology, Guangdong, China) and has-miR92a-3p inhibitors (Ruibo biotechnology, Guangdong, China) were purchased from Ribo biocompany; Mature sequence of has-miR92a-3p: UAUUGCACUUGUCCCGGCCUGU. has-miR92a-3p mimic or has-miR92a-3p inhibitor and its negative control mimics or inhibitor were transfected into HCC cells by using Lipofectamine 3000 Reagent (ThermoFisher, USA) according to the manufacturer’ instructions.

Plasmids (pcDNA3.1-E2F1, pcDNA3.1-c-Myc, pcDNA3.1-vetctor) were purchased from Repbio (Hangzhou, China). Cells were seeded into 6 cm or 10 cm dishes and incubated overnight, plasmids (2μg/6cm dish，4μg/10cm dish) were transfected into dish with HCC cells, using Lipofectamine 3000 Reagent (ThermoFisher, USA). 72 hours after transfection, cells or supernatant were collected for further RNA extraction and other applications.

**Colon formation assay**

1.0 x 10^3^ HCC per well cells were seeded into six-well plates in DMEM medium with 10% FBS and cultured for 2 weeks. Cells were washed by PBS three times and fixed by 4% paraformaldehyde for 20 min. Then we used 0.2% crystal violet to stained cells. After 30 minutes’ incubation, cells were following washed with PBS three times and counted under an optical microscope (IX82, Olympus, Japan).

**Migration and Invasion Assay**

As for migration, HCC cells (5.0 x 10^4^ cells per well) were digested and seeded into the upper chamber of Transwell plate (24-well, 8 μm pore size, Millipore, USA), cultured in 200μL DMEM without FBS. 800μL DMEM with 10% FBS were used to fulfill lower chamber. As for invasion assay, upper chambers were coated with 40μL coating medium, containing 32μL DMEM medium and 8μL Matrigel (BD Biosciences, USA), after 3 hours incubation, equal amounts of cells were seeded into the upper chamber and following were performed as above described. After certain incubation time (the migration and invasion time for Huh7 and 97h were 48h, 72h and 72h, 96h, respectively), cells were fixed by 4% paraformaldehyde and stained by 0.2% crystal violet, and the number cells were calculated.

**Antibodies**

Antibodies for western blot, immunofluorescence (IF) was listed as below: E-Cadherin (3195T, CST, USA), N-Cadherin (13116T, CST, USA), β-catenin (8480T, CST, USA), Snail (3879T, CST, USA), E2F1 (ab179445, abcam, USA), c-Myc (10828-1-AP, proteintech, China), PTEN(db986, diagbio, China), GAPDH (10494-1-AP, proteintech, China), ZO-1 (8193T, CST, USA), mTOR (2983S, CST, USA), Akt (4691S, CST, USA), phosphorylated-mTOR (5536S, CST, USA), phosphorylated-Akt (4060S, CST, USA), phosphorylated-GSK-3β (db1715, diagbio, China)

**RNA extraction and quantitative real-time polymerase chain reaction**

Total RNA was extracted by using TRIzol reagent. As for mRNA, HiScript II Q RT SuperMix (Vazyme Biotech, Nanjing, China) was used for cDNA synthesis. Expression of mRNA was measured by using SYBR Green (Vazyme Biotech, Nanjing, China). For miRNA, Mir-X™ miRNA First Strand Synthesis Kit (Takara, Kyoto, Japan) was used for cDNA synthesis. Expression of miRNA was measured by using TB Green® Premix Ex Taq™ (Tli RNase H Plus) (Takara, Kyoto, Japan). Real-time quantitative polymerase chain reaction (q-PCR) was performed in triplicate on the Bio-Rad QX100 Droplet Digital PCR system (USA). The relative expression level of target genes was calculated through normalization to GAPDH internal controls, and miRNAs were normalized with cel-miR-39 external controls. All primers were obtained from Tsingke Biological Technology (Beijng, China) and listed in the Table 2.

**Chromatin immunoprecipitation (ChIP) assay**

The ChIP Assay Kit (17-10086, Millipore, USA) was purchased and used according to the instruction of manufacturer. Briefly, cells were collected in lysis buffer and sonicated to produce DNA fragments. And lysates were incubated with antibodies anti-c-Myc (10828-1-AP, proteintech, China), anti-E2F1 (ab179445, abcam, USA) and anti-IgG, respectively. The specific binding DNA fragment (containing E2F1 binding sites and c-Myc binding sites) were extracted and purified. Specific primers were synthesized and utilized to verify the potential binding region of TFs in *miR17HG* promoter by southern blot and real time PCR.

**Immunofluorescence and in situ hybridization**

2.0 x 10^4^ 97h or Huh7 Cells per well were seeded onto confocal dishes (Thermo Fisher, MA, USA). Cells were incubated overnight with a confluence of 40%. Cells were transfected with miR-92a-3p mimics or negative control mimics. 48 hours after transfection, PBS was used to wash cells once and 4% paraformaldehyde was utilized to fixed cells at room temperature. After 30min’s fixation, cells were washed with PBS and treated with 5% BSA buffer, blocking for 1 hour. Then, cells were washed with PBS three times and incubated with 3% BSA buffer containing the first antibodies anti-E-cadherin or anti-N-cadherin overnight at 4℃. After incubation, cells were washed with PBS three times and incubated with 3% BSA buffer containing the second antibodies for 1 hour at room temperature. After incubation and washing by PBS three times, cells were stained with DAPI (4, 6-diamino-2-phenylindole) for 10 min and observed through confocal laser scanning microscopy (CLSM, IX82, Olympus, Japan).

For in situ hybridization, hsa-miR-92a-3p detection probe (5’-DIG-ACAGGCCGGGACAAGTGCAATA-DIG-3’) was purchased from Servicebio Technology company (Wuhan, China) and used to perform DAB staining according to instruction of the manufacturer. Images were captured and scanned by Pannoramic DESK, P-MIDI (3D HISTECH, Hungary) and assessed with Caseviewer software (version CV 2.3, 3D HISTECH Ltd.)

**Animal Studies**

Male nude mice (4-6 weeks old) were purchased from animal center of Zhejiang Academy of Medical Sciences and used in all experiments. All animal experiments were performed according to the procedures authorized and approved by ethical committee of the First Affiliated Hospital of Zhejiang University School of Medicine. And all animal experiments were carried out conforming to the requirement of the guidelines of the National Institutes of Health (Guide for the Care and Use of Laboratory Animals, 2011). Animals were randomly divided into different groups.

For the models to detect the effect of miR-92a-3p in the growth of HCC cells, 100 µL cell suspension (5.0 x 10^6^ cells) was subcutaneously injected into the mice. After 21days, 5 nmol antigomiR92a-3p or equivalent volume PBS was intratumorally injected every three or two days. The width and length of tumor in different time-point were measured, and the volume of tumor was calculated by the formula V=0.5*Length*Width^2^. Mice were further sacrificed in 7^th^ week, and tumors were collected for weighing and recording.

To detect the metastatic ability of HCC cells received different treatments, 100ml PBS contained 1.0 x 10^6^ 97h or 97h-luciferase cells (stable luciferase transfected 97h cells) treated with exosomes or antigomiR92a-3p were injected into mice via tail vein to construct lung metastatic models. After 8 weeks, the xenografted mice were applied to in vivo luciferase-mediated imaging. Briefly, mice were anesthetized and injected with luciferin at 150 mg/kg in a volume of 100 μL via intraperitoneal injection. Images were captured at a peak time of 15–20 min after injection using an IVIS-200 Imaging System (Xenogen Corporation, USA). The lungs of mice were collected after euthanization, and metastatic nodules was caculated. In addition, to fully determine the role of miR92a-3p in the progression of HCC, a liver metastases model was established via intra-spleen injected 97hm cells with or with the treatment of antigomiR92a-3p via tail vein, the livers of mice were collected after euthanization, and metastatic nodules was caculated.

For orthotopic liver cancer models, 100 ml PBS contained 1.0 x 106 HCC cells or 1 mm3 HCC patient derived tumor tissues were implanted into right liver lobe. After 8 weeks the xenografted mice were applied to the observation of metastatic condition and detection the level of exo-miR92a-3p in plasma.

**Author contributions**

BY, WJ and SSZ conceived the project and designed the experiments. BY, XDF, HL, JBW, HXY and CBL performed the experiments. BY, RLT and CBL analyzed and interpreted the data. BY, YHC ADN JRC drafted the paper. QYC, XLC, KJL and JW revised the manuscript. ZL, WXW, YJL, JTH, and CBL collected clinical samples and analyzed associated clinical data. The final manuscript was read and approved by all authors.

**Supplementary Figures**


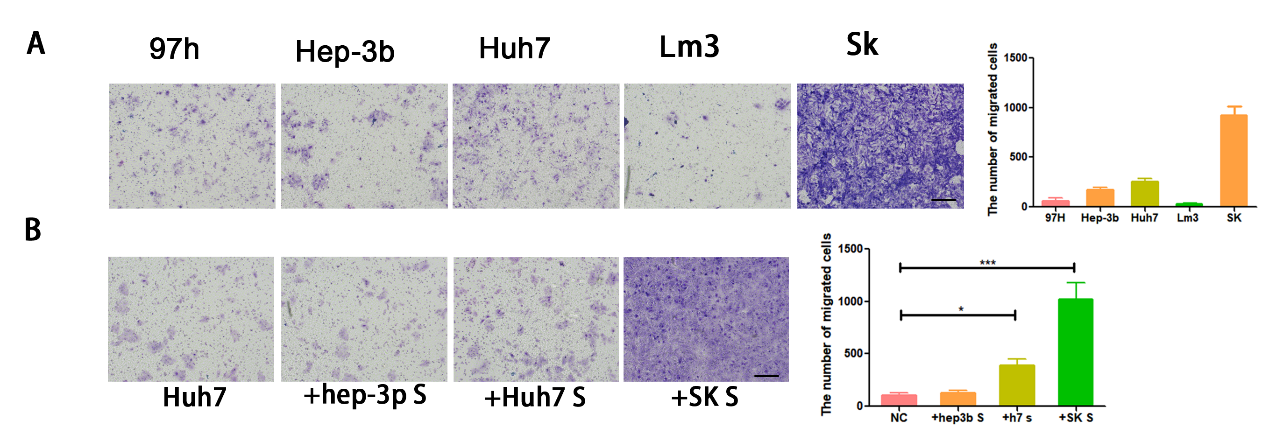


**Figure S1. Exosome derived from liver cancer cells promote migration ability of recipient cancer cells.**

Migration abilities of five liver cancer cells were detected by Transwell assay (A). Huh7 was co-cultured with the supernatant of Hep-3B, Huh7, SK, and the change of migration ability was exhibited. Migrated cells were counted and representative images were shown (B). All representative images were shown with a magnificence of 100 times. All experiments performed in triplicate, *p < 0.05; **p < 0.01; ***p < 0.001. Bars: 100nm


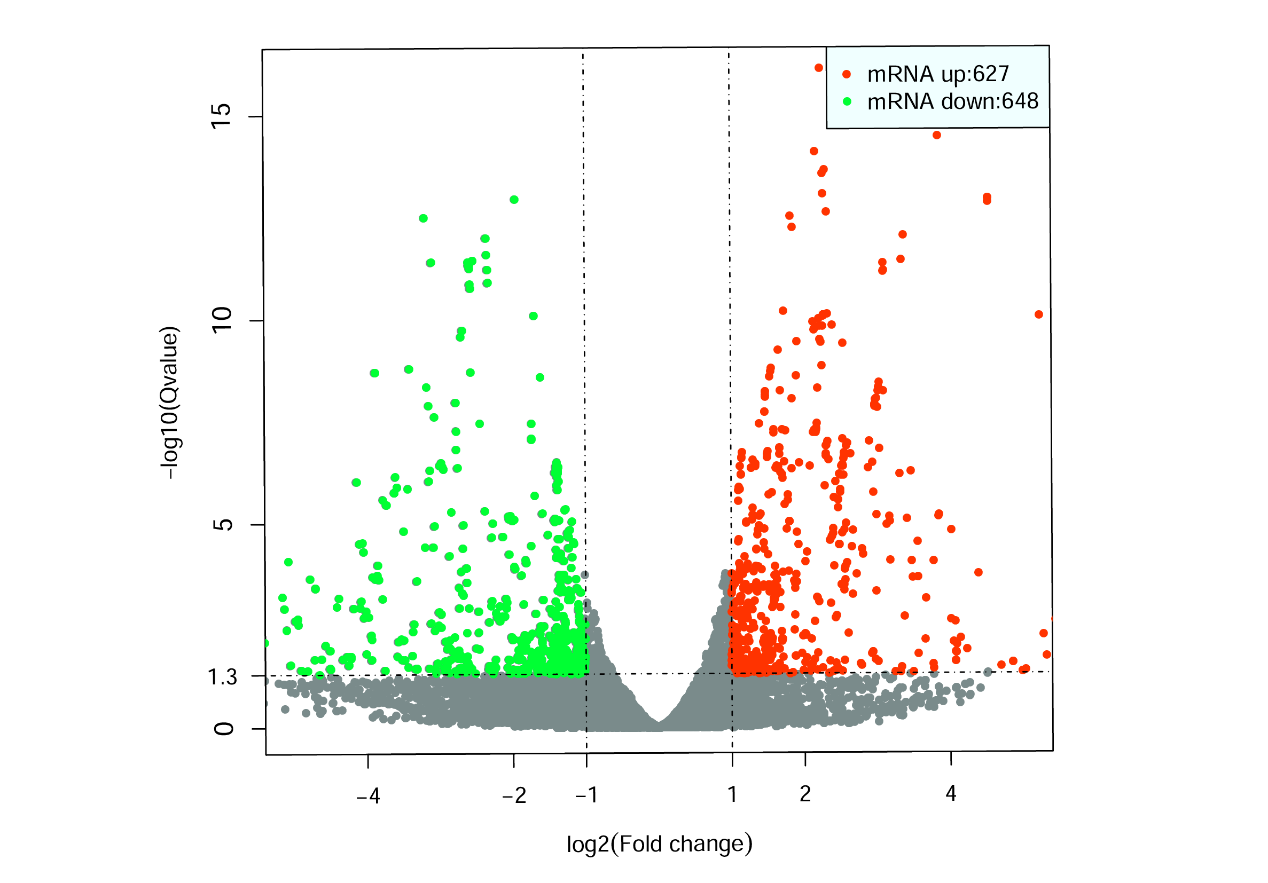


**Figure S2. miR92a-3p is overpassed in the exosomes of 97hm.**

Volcano plot of differential exosomal miRs between 97h and 97hm was shown after miRs sequencing (A)


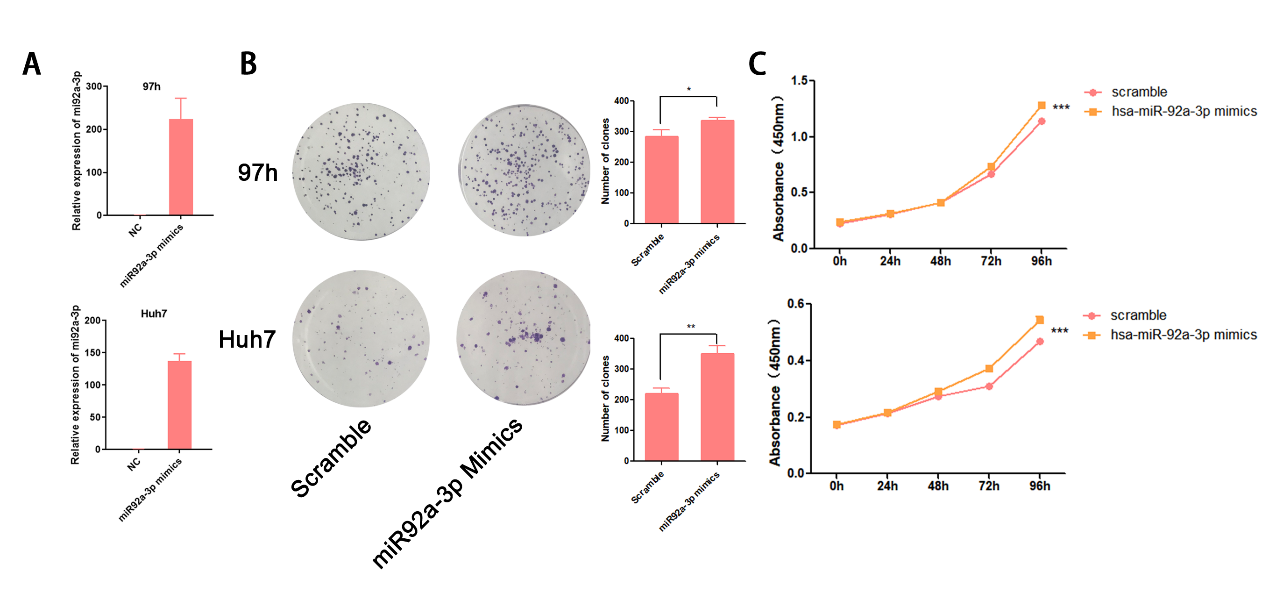


**Figure S3. MiR92a-3p promote the proliferation and cell viability of HCC cells both in vitro and vivo.**

MiR92a-3p was overexpressed by miR92a-3p mimics(A). The clone formation ability of HCC cells transfected with miR92a-3p mimics. Representative images are shown and the number of colons were calculated(B), and cell viability of HCC cell treated with miR-92a-3p mimics were represented(C), *p < 0.05; **p < 0. 01, ***p < 0. 001.


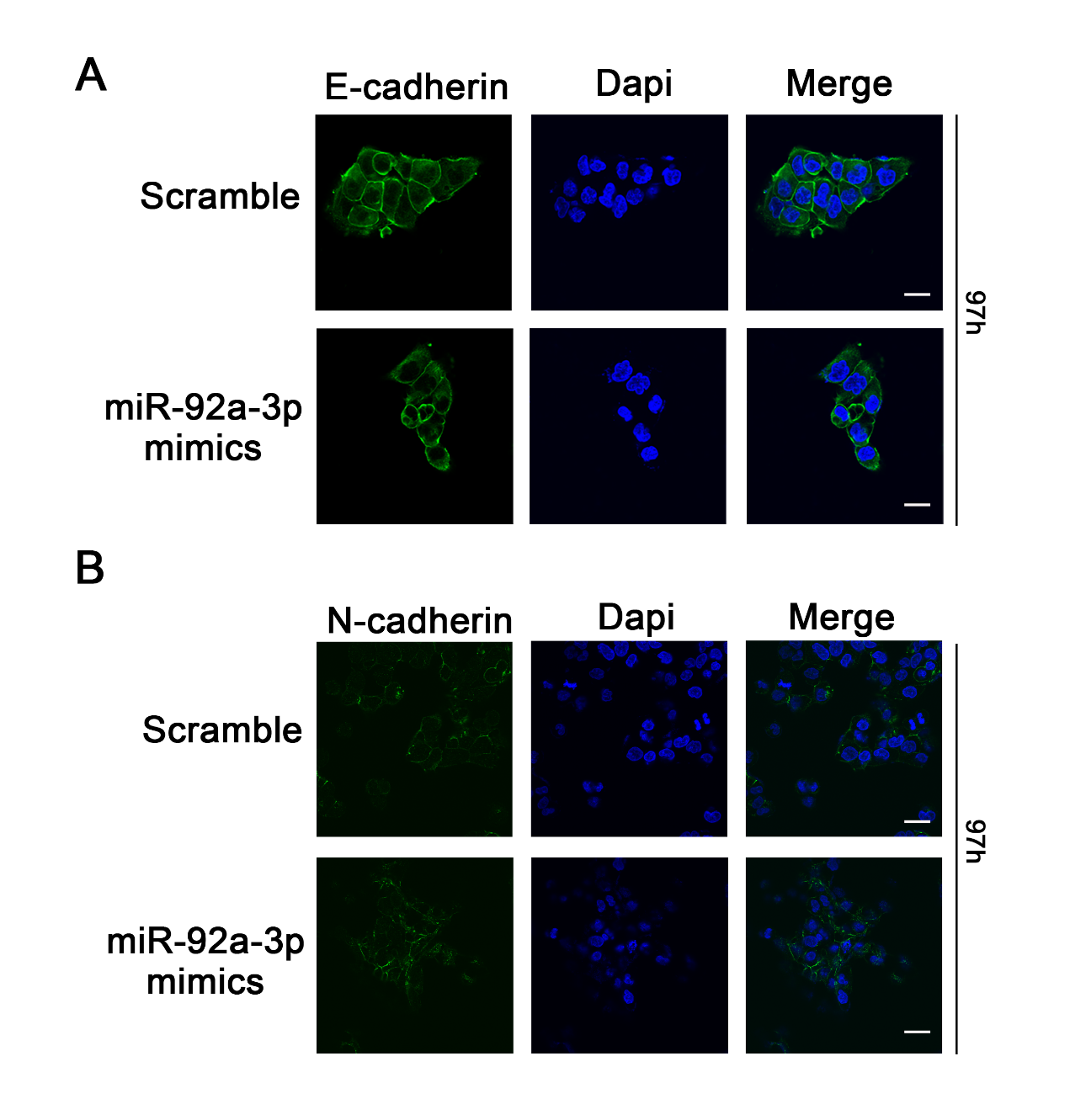


**Figure S4. MiR92a-3p facilitate epithelial-mesenchymal transition in HCC.**

Representative immunofluorescent images of E-cadherin (A) and N-cadherin (B) in 97h treated with miR92a-3p mimics and negative control mimics, with a magnification of 600 times (Blue: DAPI, Green: E-cadherin or N-cadherin). Bars: (A) 10 μm; (B) 15 μm.


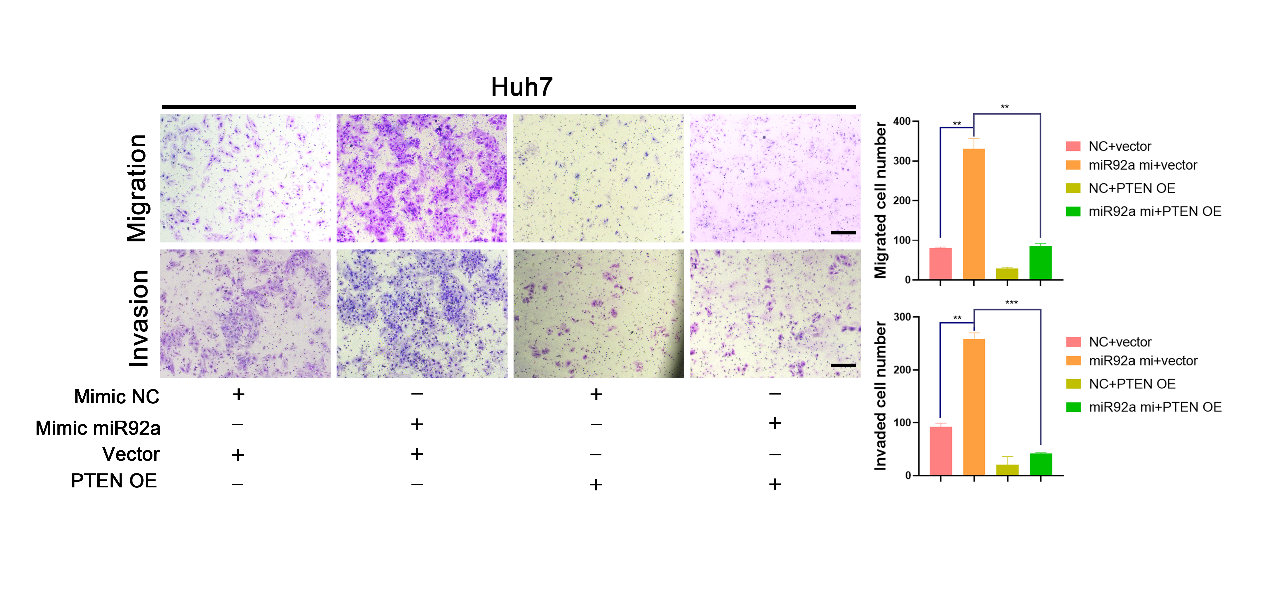


**Figure S5. PTEN overexpression ameliorate the improved metastatic ability induced by miR92a-3p in Huh7 cell.**

The results of migration and invasion assay of Huh7 cells were shown after transfected with miR92a-3p or NC mimics and PTEN overexpression or vector plasmids. The incubation time of migration assay and invasion assay are 48h and 72h respectively. *p < 0.05; **p < 0. 01, ***p < 0. 001. Bars: 100 μm.


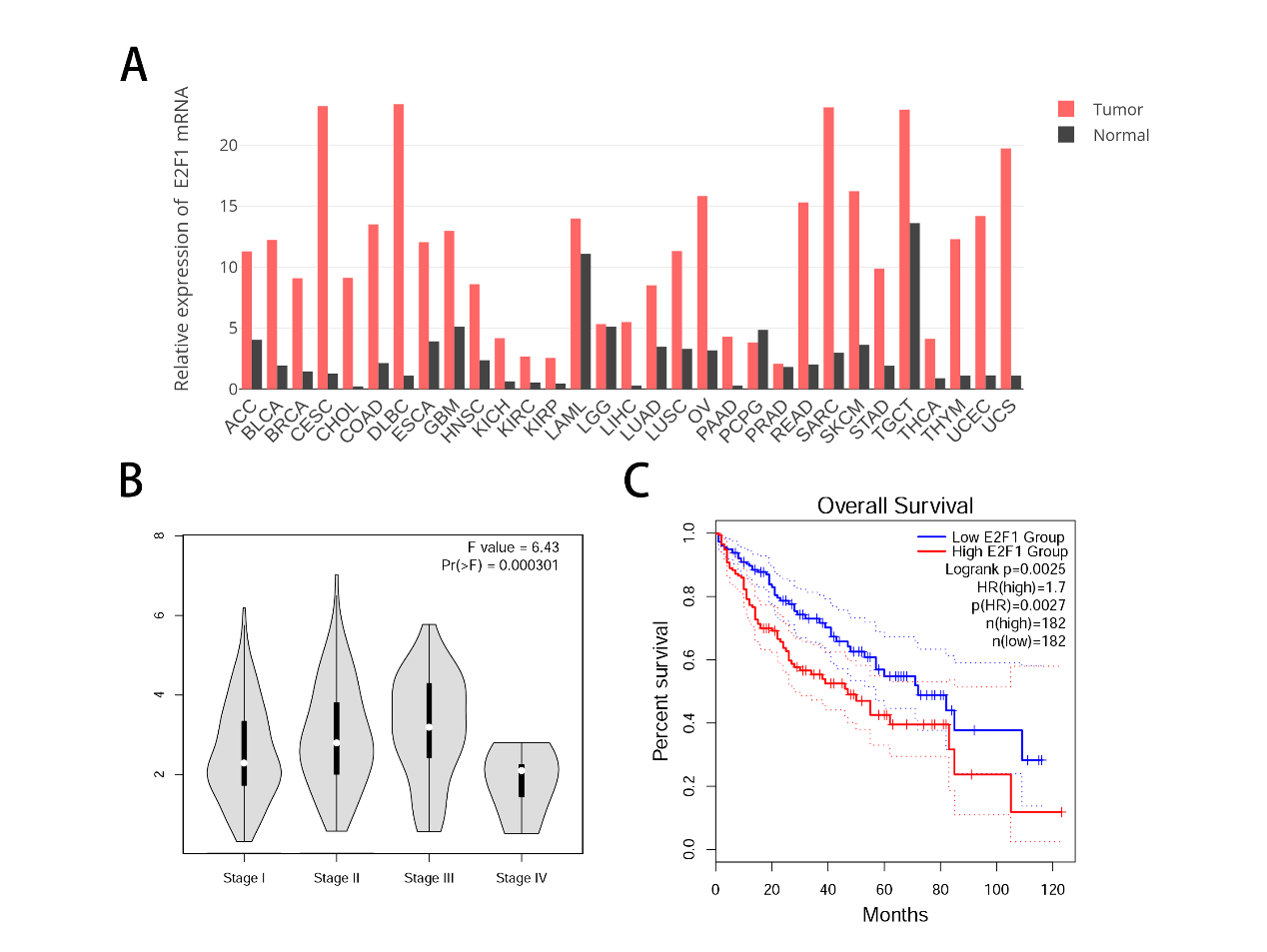


**Figure S6. E2F1 expression is correlated with prognosis of patients with HCC.**

The data from GEPIA online database was used to identify the role of E2F1 in HCC. The relative expression of E2F1 in various types of cancers and normal tissues (A). The relationship between the expression of E2F1 and TMN stage of HCC (B). The overall survival rate of HCC patients, stratified by expression of miR-92a-3p was shown, p=0.0027 (C).
